# Controlled mild water stress as a priming tool to inherent defense mechanisms of navel orange against citrus nematode, *Tylenchulus semipenetrans*

**DOI:** 10.1038/s41598-026-44988-2

**Published:** 2026-04-18

**Authors:** Atef M. El-Sagheer, Mohamed A. Saad, Ahmed M. M. Abdelghany

**Affiliations:** 1 Agricultural Zoology and Nematology Department, Faculty of Agriculture, Al-Azhar University, Assiut, 71524 Egypt; 2 Pomology Department, Faculty of Agriculture, Assiut University, Assiut, 71511 Egypt

**Keywords:** Phytonematodes, Citrus nematode management, Slow decline disease, Citrus physiological response, Drought-induced defense, Biological control, Ecology, Ecology, Physiology, Plant sciences

## Abstract

Citrus nematode, *Tylenchulus semipenetrans* Cobb, 1913 is a major economically important plant-parasitic nematode responsible for citrus slow decline worldwide. This study aimed to evaluate the efficacy of controlled mild water stress on priming defences in navel orange (*Citrus sinensis* “Navel”) seedlings and to model nematode-host interactions under stress. Five greenhouse treatments were applied: negative control (C-, 100% field capacity), positive control (C+, 100%), pre-infection water stress (T1, 40%), post-infection water stress (T2, 40%), and continuous water stress (T3, 70%). Nematode reproduction criteria, plant growth and physiological and biochemical parameters were evaluated. Results indicate that the controlled mild water stress significantly (*p* ≤ 0.05) reduced nematode reproduction. The final nematode population (Pf) and reproduction factor decreased from 6.176 to 2.47 in positive control to 2.034 and 0.81 in continuous water stress. Shoot length and fresh weight declined from 31.47 cm to 29.22 g in negative control to 7.30 cm and 6.58 g in continuous water stress. Regression based model indicated that a strong positive correlation between nematode final population and orange seedlings growth performance (R^2^ = 0.995–0.999), demonstrated that the controlled mild water stress plays a critical role in mediating the interaction between *T. semipenetrans* and its host plant. Stressed plants accumulated higher flavonoids (up to 4.563 mg QE g^− 1^ FW), phenolics (6.498 mg g^− 1^ FW min^− 1^), and proline (11.50 mg g^− 1^ FW), while chlorophyll a and b decreased to 0.607 and 0.207 mg g^− 1^ FW, respectively. These findings suggest that controlled mild water stress, particularly when applied as a pre- or post-infection, can reduce *T. semipenetrans* reproduction and enhance citrus defense mechanisms. However, continuous water stress limits plant growth, highlighting a balance between nematode suppression and host adaptation for sustainable management.

## Introduction

Plant-parasitic nematodes (Phylum: Nematoda) constitute a major biotic constraint to citrus production worldwide. Among them, the citrus nematode *Tylenchulus semipenetrans* Cobb, 1913 (Nematoda: Tylenchida) is recognized as one of the most destructive pathogens, responsible for the slow decline disease of citrus^1^. This sedentary semi-endoparasite establishes permanent feeding sites in the root cortex by partial penetration of the female body, leading to chronic root dysfunction. The citrus nematode can cause yield losses of 10–30% globally, with higher losses under favorable soil and climate conditions^2^. Citrus nematodes have been found throughout the main citrus production regions of Egypt and in a large measure jeopardize the losses of the yield and quality of the final crop. The management of *T. semipenetrans* on navel orange, *Citrus sinensis* L. Osbeck (Family: Rutaceae) the main cultivated variety of orange in Egypt by 3.7 Million tons in season of 2023–2024^3–5^.

Management of *T. semipenetrans* has traditionally relied on chemical nematicides^6^. However, increasing concerns regarding environmental pollution, pesticide residues in fruit and soil, regulatory restrictions, and the potential development of nematode resistance have substantially limited the long-term sustainability of chemical control^7^. These constraints have intensified the search for alternative, environmentally sound strategies that reduce nematode pressure while maintaining crop productivity^8^.

One promising approach is the exploitation of plant resistance mechanisms. In plant physiology, the concept of priming or induced resistance describes a state in which exposure to a mild stimulus prepares the plant for faster and stronger defense activation upon subsequent biotic stress^9^. Abiotic factors, including controlled water deficit, are known to function as effective priming agents by activating complex signaling pathways and enhancing the accumulation of defensive metabolites^10,11^. While severe drought stress negatively affects plant growth, accumulating evidence suggests that mild and transient water stress can act as a physiological elicitor rather than a damaging factor^12,13^.

Despite growing interest in priming-based strategies, little is known about the role of controlled water stress in enhancing citrus root defense specifically against *T. semipenetrans*. In particular, the extent to which mild water deficit can modify root susceptibility, nematode penetration, and establishment remains poorly understood^14,15^. This represents a critical knowledge gap, especially for citrus production systems seeking sustainable alternatives to chemical nematicides.

Therefore, the objective of this study was to evaluate the potential of controlled mild water stress as a priming strategy to enhance the defensive capacity of navel orange seedlings against *T. semipenetrans* under greenhouse conditions. We hypothesize that a transient, non-lethal water deficit acts as an early warning signal that pre-activates root defense mechanisms, thereby reducing nematode infection and development. This host-mediated approach aims to manipulate plant suitability rather than directly targeting the nematode, offering a sustainable and eco-friendly component for integrated nematode management in citrus cultivation.

## Results

### Reproduction and survival of *T. semipenetrans*

**Fig. 1:**
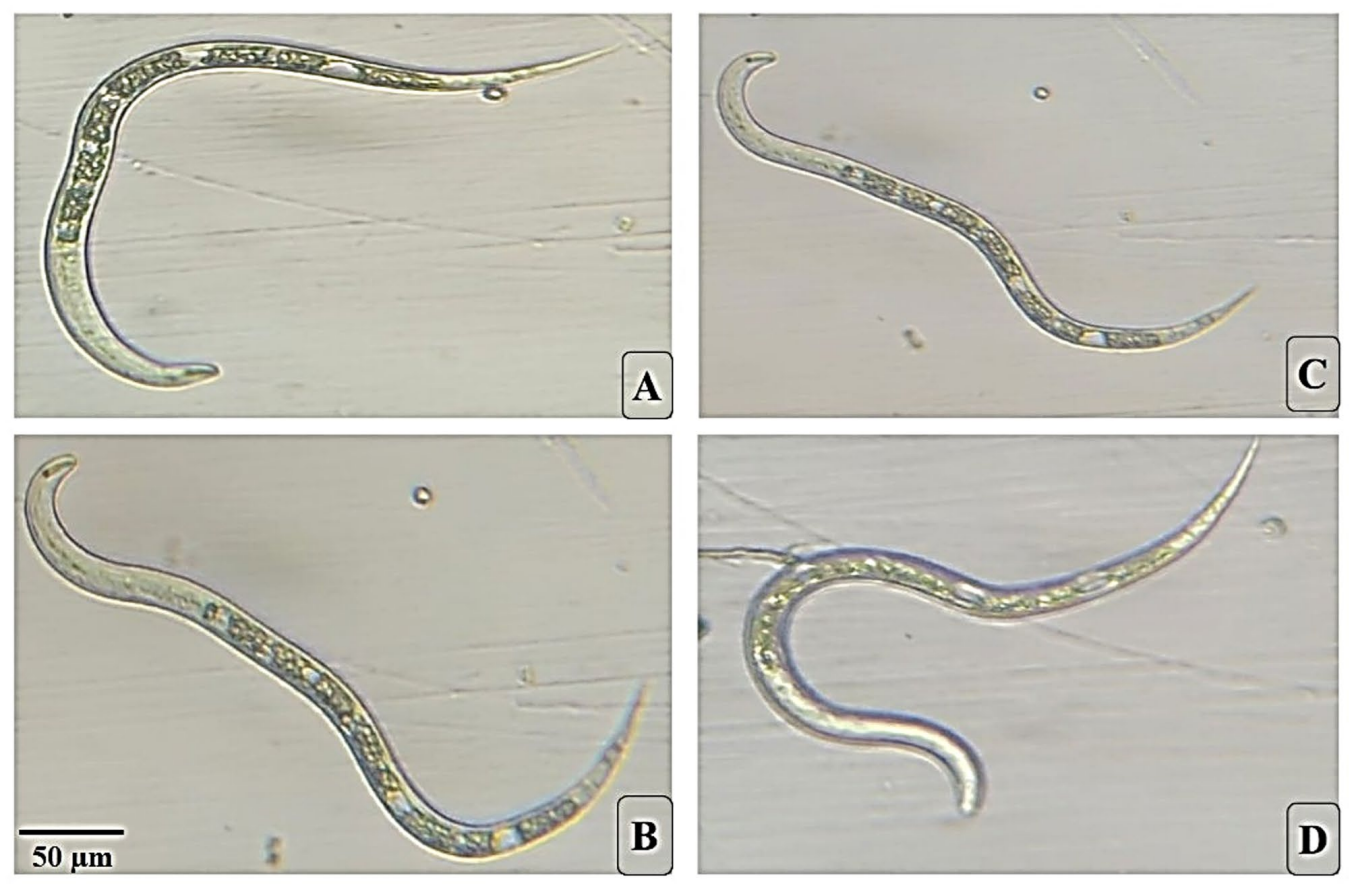
Microscopic examination of citrus nematode individuals in response to different levels of mild water stress (magnification ×100). (**A**) C+, Individuals show normal body length and curvature, high turgidity, and well-defined internal tissues. The cuticle is smooth and continuous, and the cytoplasm is uniformly distributed without any voids, indicating high physiological fitness and a healthy homeostatic state. (**B**) T1, specimens exhibit a slight reduction in body turgor and minor changes in curvature. While internal structures are largely preserved, there is the onset of internal vacuolization (visible as small, transparent circular voids). This reflects a mild stress impact and the beginning of cellular adaptation to the water-stressed environment. (**C**) T2, individual display clearer stress symptoms, including reduced turgidity and a less uniform body shape. There is a visible darkening of the internal contents and progressive internal degradation. The vacuoles have increased in size and frequency along the intestinal tract, suggesting compromised physiological performance and disrupted osmotic regulation. (**D**) T3Individual show the most pronounced stress response, characterized by an evident loss of turgor and irregular or constricted posture. This panel demonstrates severe cytoplasmic disintegration and extensive vacuolization. The internal organelles appear disorganized (cellular lysis), leading to diminished internal clarity and indicating significantly reduced vitality and fitness.

The results in Table 1; Fig. 1 demonstrate that controlled mild water stress significantly (*p* ≤ 0.05) affected the reproduction and survival of *T. semipenetrans* infecting navel orange seedlings. The number of second-stage juveniles (J2s) extracted from soil decreased progressively from the control mean of 2463 to 1729, 1376, and 1123 under pre-stress (T1), post-stress (T2), and continuous stress (T3) treatments, respectively. This reduction was highly significant (F = 474.45, R^2^ = 0.9393), reflected a strong relationship between stress intensity and nematode suppression. The number of adult females per root system followed a similar trend, declining from 55 in the control to 52, 48, and 31 in T1, T2, and T3, respectively. The difference among treatments was statistically significant, confirming that mild water stress limited female maturation.

**Table 1:** Influence of controlled mild water stress on the reproduction and survival of *T. semipenetrans* infecting navel orange seedlings under greenhouse conditions (25 ± 3 °C).

Treatments		Nematode reproduction criteria						Reduction %
		J2s in soil (250 g)	Females	Eggs	Egg mass	Pf	Rf	
C+	Positive control	2463 ± 70.93^a^	55 ± 3.61^a^	93 ± 2.00^a^	39 ± 1.53^a^	6176	2.47	-
T1	Pre-stress	1729 ± 56.52^b^	52 ± 1.63^b^	76 ± 3.06^b^	33 ± 1.15^b^	4326	1.73	29.95
T2	Post-stress	1376 ± 7.57^c^	48 ± 1.53^c^	65 ± 2.65^c^	28 ± 1.33^c^	3222	1.29	47.82
T3	Continuous stress	1123 ± 17.39^d^	31 ± 1.51^d^	46 ± 1.56^d^	19 ± 1.00^d^	2034	0.81	67.07
R^2^		0.9393	0.8371	0.9907	0.9837	0.9845	0.9845	-
F		474.45	71.67	203.67	128.32	-	-	-
LSD _0.05_		87.222	4.210	4.482	2.490	-	-	-

The negative control (C-, 100% FC) treatment was maintained nematode-free, as no inoculation was performed. Each value represents the mean of three replicates ± SD. J2s represents the second-stage juveniles extracted from soil per pot. Pf represents final population; Rf represents the reproduction factor (rate of build-up) of nematode. Eggs represents the mean of five random egg-masse peer root. R^2^ represents the coefficient of determination, indicating the strength and direction of the relationship between the control and treated values. Values followed by the same letter (s) in a column do not significantly differ according to according to Duncan’s multiple-range test (*p* ≤ 0.05). LSD values represent the level of significance (*P* ≤ 0.05) between treatments according to Duncan’s multiple-range test.

Egg production per egg mass also decreased significantly with increasing stress intensity, with means of 93, 76, 65, and 46 for C+, T1, T2, and T3, respectively. The number of egg masses per root exhibited a comparable reduction trend, dropping from 39 in the control to 33, 28, and 19 under the same sequence of treatments. The Pf followed a clear descending pattern among treatments. The highest value was recorded in C+ (6176), while a significant decrease was observed in T1 treatment (4326). The Pf declined further in T2 (3222) and reached its lowest level under continuous stress T3 (2034). A similar pattern was obtained for the reproduction factor (Rf), which was highest in C+ (2.47), then decreased in T1 (1.73) and T2 (1.29), attaining its minimum in T3 (0.81). In addition, the high coefficients of determination (R^2^ = 0.8371 to 0.9907) confirmed the strong linear association between increasing water-stress intensity and the reduction in nematode reproduction and survival.

Moreover, the least significant difference (LSD _0.05_) values indicate that all nematode reproduction criteria including J2s in soil, females, eggs, and egg masses (87.222, 4.210, 4.482 and 2.490) respectively, were differed significantly among treatments at the 5% probability level. Those low values showed that the reductions recorded under the different water stress levels were statistically distinct and not due to random variation.

### Vegetative growth

The vegetative growth of navel orange seedlings was significantly influenced by the interactive effects of tested water stress and infection with *T. semipenetrans* (Tables 2 and 3). All measured growth parameters; shoot length (SL), shoot and root biomass (fresh and dry weights), root length (RL), and leaf relative water content (LRWC), showed revealed highly significant differences (*p* ≤ 0.05) reductions with increasing water stress intensity. Shoot length was greatest in the negative control (C-) (31.47 cm) and significantly reduced in the positive control (C+) (19.80 cm). Further declines were observed in the pre-stress (T1) (14.20 cm), post-stress (T2) (11.80 cm), and continuous stress (T3, 7.30 cm) treatments. The reduction was highly significant, confirming a strong negative relationship between stress intensity and shoot elongation.

**Table 2:** Interactive effects of controlled mild water stress on shoot traits of navel orange seedlings infecting with *T. semipenetrans* under greenhouse conditions (25 ± 3 °C).

Treatments		Shoot Traits			TPFW (g)	TPL (cm)
		SL (cm)	SFW (g)	SDW (g)		
C-	Negative control	31.47 ± 0.74^a^	29.22 ± 0.30^a^	9.84 ± 0.11^a^	34.343	55.767
C+	Positive control	19.80 ± 0.46^b^	21.33 ± 0.37^b^	6.72 ± 0.10^b^	23.207	36.833
T1	Pre-stress	14.20 ± 0.30^c^	15.61 ± 0.79^c^	6.36 ± 0.46^c^	17.133	28.700
T2	Post-stress	11.80 ± 0.44^d^	10.49 ± 0.37^d^	4.50 ± 0.20^d^	11.877	25.200
T3	Continuous stress	7.30 ± 0.46^e^	6.58 ± 0.17^e^	3.60 ± 0.25^e^	7.780	19.633
R^2^		0.917	0.9832	0.9316	-	-
F		1043.73	1180.0	253.64	-	-
LSD _0.05_		0.907	0.820	0.476	-	-

Each value represents the mean of three replicates ± SD. SL represents shoot length, SFW represents shoot fresh weight, SDW represents shoot dry weight, RL represents root length, RFW represents root fresh weight (g), RDW represents root dry weight, LRWC represents leaf relative water content, TPFW represents total plant fresh weight, and TPL represents total plant length. R² represents the coefficient of determination, indicating the strength and direction of the relationship between the control and treated values. Values followed by the same letter (s) in a column do not significantly differ according to according to Duncan’s multiple-range test (*p* ≤ 0.05). LSD values represent the level of significance (*P* ≤ 0.05) between treatments according to Duncan’s multiple-range test.

**Table 3:** Interactive effects of controlled mild water stress on root traits of navel orange seedlings infecting with *T. semipenetrans* under greenhouse conditions (25 ± 3 °C).

Treatments		Root Traits			
		RL (cm)	RFW (g)	RDW (g)	LRWC (%)
C-	Negative control	24.30 ± 0.53^a^	5.13 ± 0.45^a^	3.71 ± 0.28^a^	89.71 ± 0.87^a^
C+	Positive control	17.03 ± 0.25^b^	1.87 ± 0.06^b^	1.58 ± 0.09^b^	84.03 ± 0.81^b^
T1	Pre-stress	14.50 ± 0.30^c^	1.52 ± 0.11^c^	1.30 ± 0.07^c^	68.77 ± 0.86^c^
T2	Post-stress	13.40 ± 0.46^d^	1.38 ± 0.22^d^	0.88 ± 0.10^d^	56.45 ± 2.47^d^
T3	Continuous stress	12.33 ± 0.15^e^	1.20 ± 0.11^e^	0.23 ± 0.10^e^	47.31 ± 1.72^e^
R^2^		0.8267	0.644	0.8479	0.9847
F		517.0575	145.203	171.542	429.714
LSD _0.05_		0.664	0.429	0.265	2.721

Each value represents the mean of three replicates ± SD. SL represents shoot length, SFW represents shoot fresh weight, SDW represents shoot dry weight, RL represents root length, RFW represents root fresh weight (g), RDW represents root dry weight, LRWC represents leaf relative water content, TPFW represents total plant fresh weight, and TPL represents total plant length. R² represents the coefficient of determination, indicating the strength and direction of the relationship between the control and treated values. Values followed by the same letter (s) in a column do not significantly differ according to according to Duncan’s multiple-range test (*p* ≤ 0.05). LSD values represent the level of significance (*P* ≤ 0.05) between treatments according to Duncan’s multiple-range test.

A similar pattern was recorded for shoot fresh weight (SFW), which decreased from 29.22 g in C- to 21.33 g in C+, 15.61 g in T1, 10.49 g in T2, and 6.58 g in T3. The corresponding dry weight (SDW) declined steadily from 9.84 g in the control to 6.72, 6.36, 4.50, and 3.60 g in the same sequence of treatments, with a highly significant F-value (253.64) and R^2^ = 0.9316, indicating a strong linear association between the level of stress and biomass reduction. Root length (RL) followed the same descending pattern, ranging from 24.30 cm in C- to 17.03 cm in C+, and dropping further to 14.50, 13.40, and 12.33 cm under T1, T2, and T3, respectively. Similarly, root fresh weight (RFW) and root dry weight (RDW) showed continuous decreases from 5.13 g to 3.71 g in C- to 1.87 g and 1.58 g in C+, reaching their lowest values of 1.20 g and 0.23 g under T3. These reductions were statistically significant (R^2^ = 0.644 for RFW and 0.8479 for RDW). Leaf relative water content (LRWC) also declined sharply with stress application, dropping from 89.71% in C- to 84.03% in C+, and further to 68.77%, 56.45%, and 47.31% under T1, T2, and T3, respectively. The high F-value (429.714) and R^2^ (0.9847) reflected a strong dependence of leaf hydration status on water availability. Furthermore, the consistently high coefficients of determination (R^2^ = 0.64 to 0.98) across all parameters indicate a strong negative linear relationship between water stress and vegetative performance of nematode-infected citrus seedlings. In addition, LSD _0.05_ values for all vegetative growth parameters; SL, SFW, RFW, SDW, RDW, RL and LRWC (0.907, 0.820, 0.476, 0.664, 0.429, 0.265, and 2.721), respectively indicate that significant differences existed among treatments at the 5% probability level, and not attributed to random variation.

### Interaction of *T. semipenetrans* with vegetative growth

**Fig. 2:**
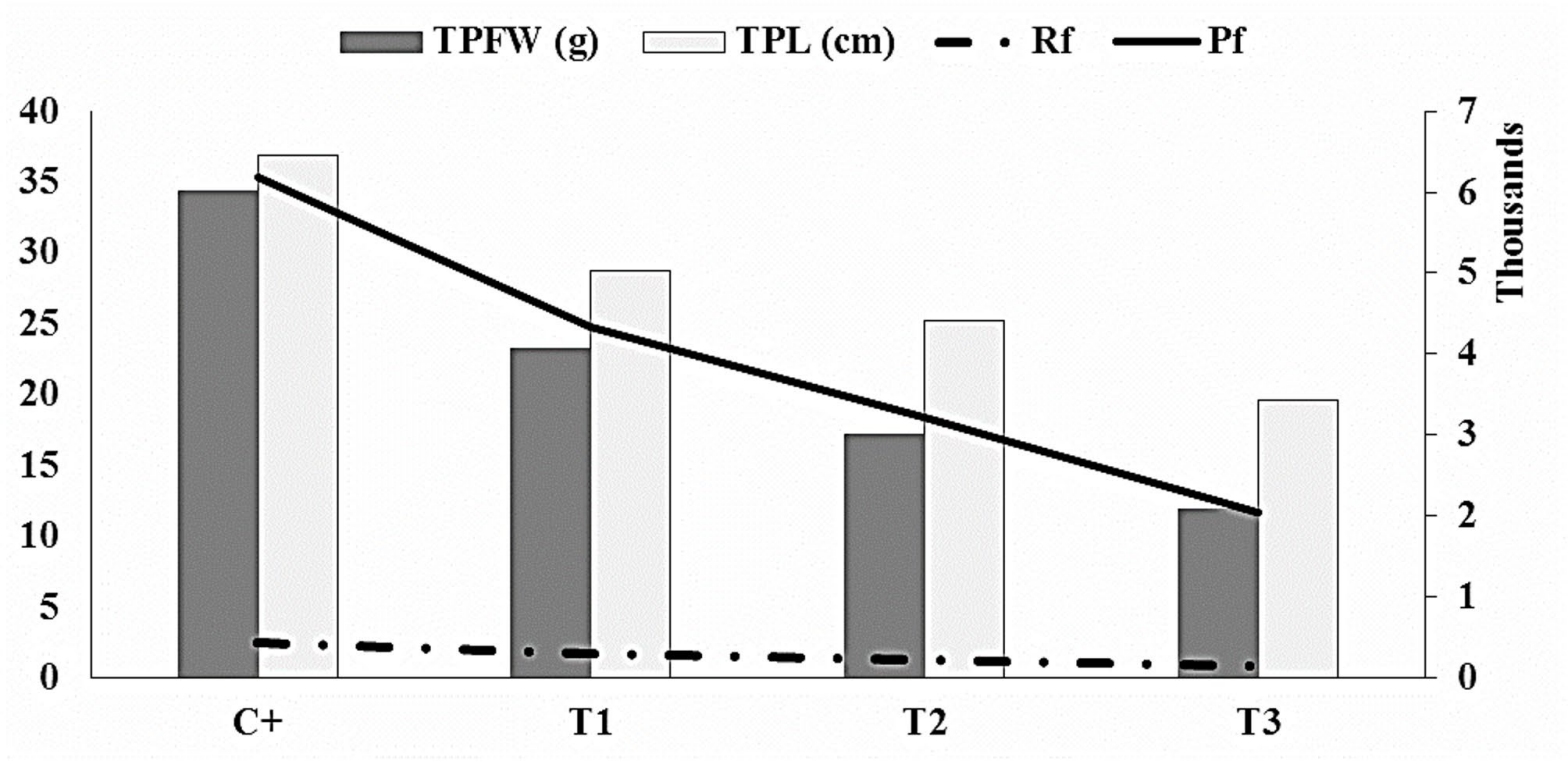
Integrated regression-based matrix illustrating the interaction between *T. semipenetrans* and orange seedling growth parameters. The model demonstrates a strong positive correlation between the nematode final population (Pf) (right Y-axis), reproduction factor (Rf) and plant growth parameters (left Y-axis) (total plant fresh weight, TPFW; and total plant length, TPL), suggesting that controlled mild water stress significantly influences the relationship between *T. semipenetrans* and navel orange seedlings.

To evaluation the interaction between nematode population dynamics and navel orange seedlings performance under controlled mild water stress levels under greenhouse conditions, a regression-based model (integrated matrix) was used to integrate *T. semipenetrans* and plant growth parameters. The data presented in Table 4; Fig. 2 indicated that a strong positive correlation between nematode final population and orange seedlings growth performance, suggesting that mild water stress plays a critical role in mediating the interaction between *T. semipenetrans* and its host plant.

**Table 4:** Integrated matrix to evaluation of the interaction between *T. semipenetrans* criteria and navel orange seedlings growth parameters with regression model outputs under greenhouse conditions (25 ± 3 °C).

Treatment		Pf	Rf	TPFW (g)	TPL (cm)	Fit model equation	*R*²	*p*-value (s)
C+	Positive control	6176	2.47	34.343	36.833	TPFW = 0.13 + 13.65×Rf	0.9950	0.00247 (Rf)
T1	Pre-stress	4326	1.73	23.207	28.700	TPFW = 0.09 + 0.00547×Pf	0.9954	0.00232 (Pf)
T2	Post-stress	3222	1.29	17.133	25.200	TPFW = − 0.83 + 0.133×Pf − 319.47×Rf	0.9988	0.321 (Pf) 0.333 (Rf)
T3	Continuous stress	2034	0.81	11.877	19.633	TPL = 11.93 − 0.0557×Pf + 149.29×Rf	0.9978	0.597 (Pf) 0.575 (Rf)

The negative control (C-, 100% FC) treatment was maintained nematode-free, as no inoculation was performed. Pf represents final population, Rf represents the reproduction factor (rate of build-up) of nematode. TPFW represents total plant fresh weight, and TPL represents total plant length. Fit model equation represents the regression relationship that most accurately describes the interaction between nematode.

In the control treatment, the Pf reached 6,176 individuals, with a reproduction factor (Rf) of 2.47. Plants grown under this condition exhibited the highest growth performance, recording a total plant fresh weight (TPFW) of 34.34 g and a total plant length (TPL) of 36.83 cm. Under pre- and post-stress conditions, both Pf and Rf values declined moderately (4,326, 3,222) and (1.73, 1.29) respectively, accompanied by noticeable reductions in TPFW (23.21 and 17.13 g) and TPL (28.70 and 25.20 cm). The lowest nematode reproduction and poorest plant growth were observed under continuous stress, where Pf dropped to 2,034 and Rf to 0.81, while plant fresh weight and length decreased to 11.88 g and 19.63 cm, respectively.

Regression analyses confirmed strong correlations between nematode parameters and plant growth indicators. The simple linear regression between Rf and TPFW (R^2^ = 0.9950, *p* = 0.00247) and between Pf and TPFW (R^2^ = 0.9954, *p* = 0.00232) showed highly significant relationships, indicating that nematode reproductive potential is closely linked to plant biomass production. Multiple regression models combining Pf and Rf as predictors improved the coefficient of determination (R^2^ up to 0.9988). Similarly, the model relating plant length (TPL) to Pf and Rf (R^2^ = 0.9978).

### Physiological and biochemical responses

To evaluate the biochemical basis of the citrus seedlings defensive response against *T. semipenetrans* infection under greenhouse conditions, a set of key physiological and biochemical indicators the non-enzymatic antioxidants (total flavonoids and total phenolics), osmolytes acting as stress osmoprotectants (proline), enzymatic antioxidants associated with oxidative stress regulation (peroxidase, polyphenol oxidase, and catalase), and photosynthetic pigments (chlorophyll a and b) was assessed.

**Fig. 3:**
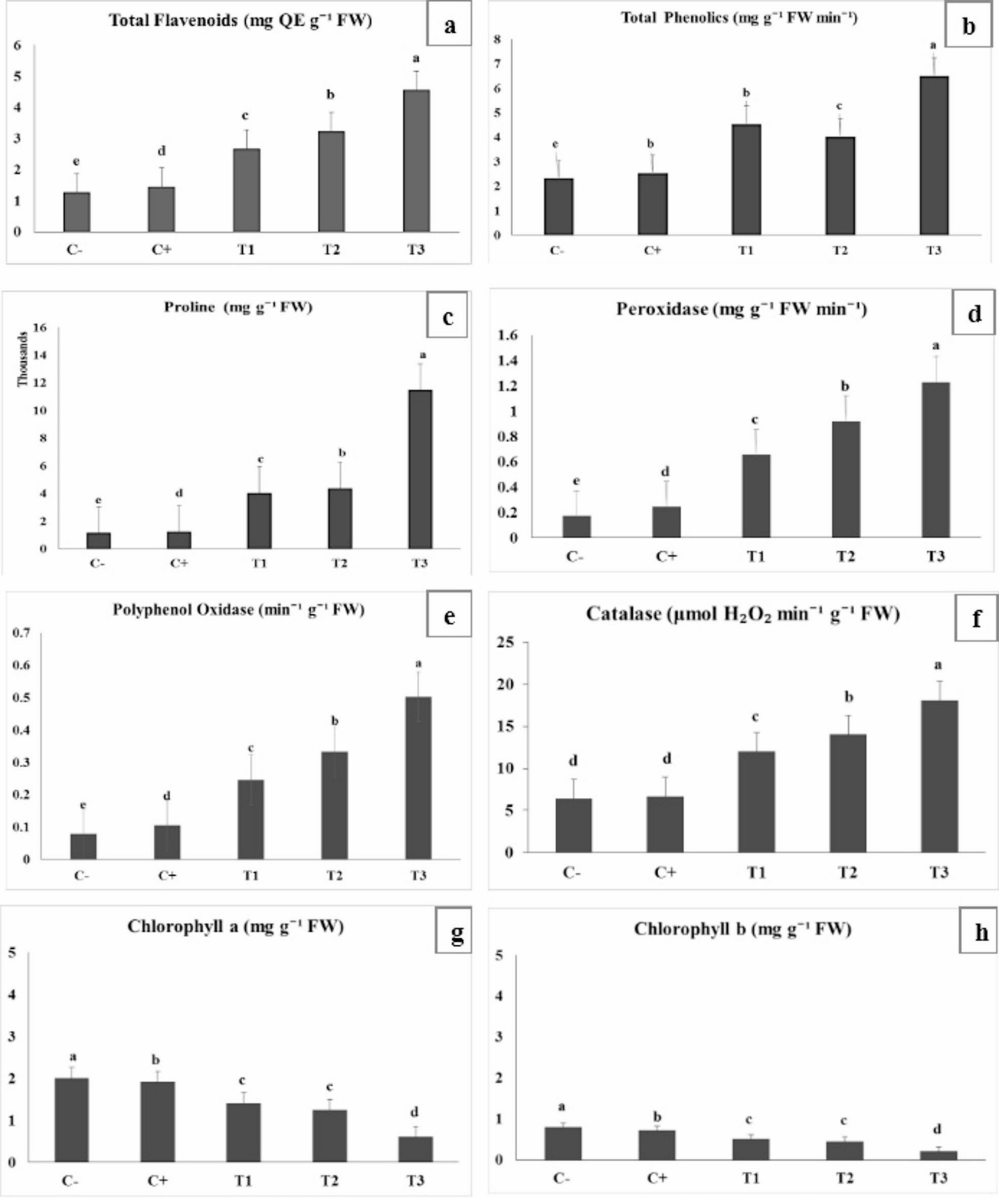
Physiological and biochemical response of navel orange seedlings infecting with *T. semipenetrans* under controlled mild water stress under greenhouse conditions (25 ± 3 °C). Each value represents the mean of three replicates. Values followed by the same letter (s) in a column do not significantly differ according to according to Duncan’s multiple-range test.

Data presented in Fig. 3 resulted in significant (*p* ≤ 0.05) physiological and biochemical modifications in orange seedlings. Which indicates the integrated impact of nematode parasitism and mild water stress intensity, through a consistent progression in antioxidant and osmoprotectant accumulation with increasing stress intensity, counterbalanced by a steady decline in photosynthetic pigments. In the negative control (C-), plants maintained balanced metabolic activity, as indicated by the modest levels of total flavonoids (1.269 mg QE g^− 1^ FW) and total phenolics (2.320 mg g^− 1^ FW min^− 1^). Proline accumulation was relatively low (1161.195 mg g^− 1^ FW), and the enzymatic antioxidant system remained at baseline, with POD (0.173 mg g^− 1^ FW min^− 1^), PPO (0.079^− 1^ g^− 1^ FW), and CAT (6.400 µmol H₂O₂ min^− 1^ g^− 1^ FW). The photosynthetic pigments were highest in this treatment, with chlorophyll a (2.001 mg g^− 1^ FW) and chlorophyll b (0.803 mg g^− 1^ FW). Once infection was introduced in the positive control (C+), plants exhibited early biochemical stress responses, including slight increases in total flavonoids (1.455 mg QE g^− 1^ FW) and phenolics (2.523 mg g^− 1^ FW min^− 1^). Proline accumulation (1260.901 mg g^− 1^ FW) and elevated antioxidant enzyme activities; POD (0.247), PPO (0.106), and CAT (6.673) suggested a mild oxidative challenge triggered by nematode invasion. Meanwhile, chlorophyll a (1.915 mg g^− 1^ FW) and b (0.725 mg g^− 1^ FW) slightly declined, reflecting an initial disruption in photosynthetic efficiency. Under pre-stress conditions (T1), biochemical defense mechanisms intensified considerably. The synthesis of total flavonoids rose to 2.678 mg QE g^− 1^ FW, and phenolic content nearly doubled (4.529 mg g^−1^FW min^− 1^). A substantial proline build-up (4032.818 mg g^− 1^ FW) marked enhanced osmotic regulation, while antioxidant enzymes became highly active; POD (0.656), PPO (0.246), and CAT (12.017). Simultaneously, chlorophyll a and b declined to 1.409 and 0.511 mg g^− 1^ FW, respectively, suggesting early pigment degradation due to stress-induced ROS formation. In the post-stress treatment (T2), these responses became more pronounced. Total flavonoids increased further to 3.234 mg QE g^− 1^ FW, while total phenolics stabilized around 4.012 mg g^− 1^ FW min^− 1^. Proline content reached 4371.149 mg g^− 1^ FW, supporting its role in osmotic adjustment and membrane stabilization. The antioxidant system remained highly active, as POD (0.922), PPO (0.333), and CAT (14.021) continued to rise. Conversely, photosynthetic pigment levels dropped further chlorophyll a (1.250 mg g^− 1^ FW) and b (0.450 mg g^− 1^ FW) indicating sustained oxidative damage to chloroplasts. The continuous stress treatment (T3) induced the strongest biochemical and physiological responses. Flavonoid content reached its peak (4.563 mg QE g^− 1^ FW), while total phenolics accumulated substantially (6.498 mg g^− 1^ FW min^− 1^). Proline content exhibited a remarkable increase to 11491.667 mg g^− 1^ FW, confirming a critical osmotic defense state. Antioxidant enzymes showed their highest activities, with POD (1.230 mg g^− 1^ FW min^− 1^), PPO (0.503^− 1^ g^− 1^ FW), and CAT (18.093 µmol H₂O₂ min^− 1^ g^− 1^ FW). Meanwhile, the pronounced decline in chlorophyll a (0.607 mg g^− 1^ FW) and b (0.207 mg g^− 1^ FW) revealed severe impairment of the photosynthetic machinery.

Overall, all tested stressed treatments differed significantly from the controls, exhibiting a uniform ascending order for antioxidant and osmolyte parameters, and an inverse pattern for chlorophyll content. This reciprocal relationship underscores the adaptive trade-off between enhanced stress defence and reduced photosynthetic capacity in Citrus under the combined impact of nematode infection and water stress.

## Discussion

The present experiment was conducted under greenhouse conditions to evaluate the efficacy of mild water stress in priming defenses of navel orange seedlings to citrus nematode *T. semipenetrans* control with water management. Through eco-physiological strategies that could contribute to the development of sustainable nematode management programs in citrus production systems.

The observed reduction in nematode population density and reproduction factor under water stress suggests that host suitability is strongly influenced by stress-mediated changes in root function and rhizosphere conditions^16^. Altered cell turgor, reduced nutrient flux, and modified root exudation patterns under limited water availability may collectively constrain nematode feeding efficiency and reproductive success, as previously reported for other sedentary and migratory nematodes under moderate drought scenarios^17,18^.

In addition to physical and nutritional limitations, stress-induced biochemical adjustments in roots may play a regulatory role in nematode suppression. The accumulation of secondary metabolites under mild water stress conditions has been linked to enhanced basal resistance against soil-borne pathogens, including nematodes, through deterrent or inhibitory effects on penetration and development^19^. In this context, the reduced nematode performance observed in moderately stressed plants likely reflects an indirect consequence of host metabolic reprogramming rather than a direct nematicidal effect of water stress itself^20^.

Plant growth reduction under combined nematode infection and water limitation reflects the additive physiological burden imposed by biotic and abiotic stressors. Declines in shoot and root biomass, organ length, and relative water content indicate that nematode parasitism amplifies the cost of water deficit by impairing root hydraulic conductivity and increasing internal water imbalance^21^. Similar growth penalties have been reported in potato infected with nematodes, where reductions in biomass and hydration status were associated with enhanced oxidative stress markers, including malondialdehyde and hydrogen peroxide^22^. These responses are consistent with broader evidence that nematode-induced tissue disruption accelerates oxidative imbalance and compromises membrane integrity, thereby limiting plant capacity for sustained growth under water deficit^23^.

Nevertheless, the partial maintenance of vegetative growth under moderate stress treatments (T1 and T2) indicates the activation of compensatory physiological mechanisms that mitigate stress damage. Such responses may involve improved water-use efficiency, selective resource allocation, or enhanced antioxidant buffering capacity, as suggested in citrus and other perennial crops exposed to transient or mild stress conditions^24,25^. These findings emphasize that stress intensity and duration critically determine whether defense activation translates into functional tolerance or growth suppression.

The pronounced increase in antioxidant metabolites and enzymes under nematode and water stress reflects a coordinated response to elevated reactive oxygen species (ROS) production. The accumulation of phenolics and flavonoids contributes to ROS scavenging and structural reinforcement of cell walls, which may indirectly restrict nematode establishment^26^. Proline accumulation further supports osmotic adjustment and redox balance, enhancing membrane stability under combined stress conditions^27^. Concurrent increases in POD, PPO, and CAT activities indicate an enzymatic reinforcement of oxidative stress mitigation pathways, consistent with observations in citrus and other crops infected with sedentary nematodes^28,29^.

In contrast, the progressive decline in chlorophyll a and b contents with increasing water stress reflects the vulnerability of the photosynthetic apparatus to prolonged oxidative pressure. Chlorophyll degradation under combined stress conditions is commonly associated with impaired carbon assimilation and reduced energy availability, potentially constraining both growth and defense metabolism^30,31^. The relatively higher chlorophyll retention observed in pre- and post-stress treatments suggests that moderate stress exposure allows plants to activate protective antioxidant systems without inducing irreversible damage to chloroplast structures, highlighting a critical threshold beyond which stress becomes detrimental rather than adaptive^32^.

Overall, the interaction between nematode infection and water stress reveals a non-linear physiological response, where moderate stress promotes defense-related metabolic adjustments, while continuous stress leads to metabolic exhaustion and functional decline^20,33^. Although this response pattern is consistent with the conceptual framework of hormesis, the present findings should be interpreted as physiological trends rather than definitive experimental validation of hormetic mechanisms^34,35^.

## Conclusion

The current study demonstrates that controlled mild water stress can act as an ecological tool to strengthen plant defence and reduce nematode infection pressure in citrus production systems. The combined enhancement of antioxidant metabolism, osmolyte accumulation, and reduced nematode reproduction supports the potential for integrating such physiological manipulations into sustainable citrus nematode management frameworks. Among the tested treatments, pre-infection and post-infection mild water stress appear most promising for integrated management, as they moderately suppress nematode reproduction while maintaining reasonable plant growth. In practical field applications, careful monitoring of irrigation and soil moisture is essential to implement such priming regimes effectively. Future research should focus on optimizing stress thresholds and irrigation schedules to achieve the desired balance between plant defense activation and productivity, potentially coupled with biological control agents or organic amendments for a comprehensive eco-friendly management system.

## Methods

### Source of *T. semipenetrans* inoculum

A population of *T. semipenetrans* was established from an inoculum collected from the rhizosphere of mature sweet orange, *Citrus sinensis* L. Osbeck trees, exhibiting characteristic symptoms of slow decline, in a commercial orchard located in the Sahel Selim province, Assiut Governorate, Egypt. Soil and root samples were transported to the laboratory for nematode extraction. Second-stage juveniles (J2s) were extracted from the soil using a combination of sieving and a modified Baermann funnel technique^36^. Actively motile juveniles collected in the water were concentrated, identified morphologically to species level based on description keys of Thorne (1962)^37^, and counted under a stereomicroscope by Hawksley counting slide. The resulting J2s suspension was adjusted with distilled water to a density of 2,500 J2s per 5 ml for subsequent inoculation.

### Plant material and growth conditions

The trial was conducted at natural growing of 2024 (February to March) in a greenhouse under moderate temperature conditions (25 ± 3 °C), natural light, and daylight period approximately 11 to 13 h. Uniform, six-month-old seedlings of navel orange (grafted onto a commonly used citrus rootstock: sour orange, *Citrus aurantium* L.) were used. Each seedling was transplanted into a 3-kg plastic pot containing a sterilized sandy loam soil mixture (1:3 v/v, autoclaved at 120 °C for 2.5 h) containing 1.2% organic matter. The soil had a pH of 7.85, with an electrical conductivity (EC) of 2.87 dS m^− 1^ and a saturation percentage of 20%. Chemical analysis of the soil saturation extract revealed soluble cations including sodium (26.0 meq L^− 1^), calcium (11.10 meq L^− 1^), magnesium (7.10 meq L^− 1^), and potassium (0.29 meq L^− 1^). The soluble anions comprised chloride (41.43 meq L^− 1^), sulfate (16.37 meq L^− 1^), bicarbonate (7.30 meq L^− 1^), and carbonate (0.26 meq L^− 1^)^38^.

## Experimental design and treatments

The pots were arranged in a completely randomized design (CRD), with five treatments and three replications (one pot with one seedling for each replicate) per treatment. The treatments were defined as follows:

C^−^ (Negative control) = (optimum): Regular irrigation at 100% of field capacity (no water stress) + no nematodes.

C^+ (^Positive control) = Regular irrigation at 100% of field capacity + with nematodes.

T1 = Mild pre-infection stress: Apply mild water stress (irrigation at 40% of field capacity) for 14 days before nematode inoculation, then return to optimal irrigation during and after inoculation.

T2 = Mild post-infection stress: Apply mild water stress (irrigation at 40% of field capacity) for 14 days immediately after nematode inoculation, then return to optimal irrigation.

T3 = Mild continuous stress: Continuous irrigation at 70% of field capacity throughout the experiment (before and after inoculation).

### Inoculation and irrigation management

An acclimatization period (two weeks) was provided for all orange seedlings prior to treatment initiation to ensure uniform growth and adaptation to greenhouse conditions under optimal irrigation. A balanced fertilizer (NPK 12-12-12) was applied uniformly to all treatments to ensure adequate nutrient availability. For *T. semipenetrans* inoculation, each pot assigned to the C+, T1, T2, and T3 treatments were inoculated with 2500 J2s of *T. semipenetrans*, which were introduced into three holes (5 cm deep) around the base of each seedling. The negative control (C-) received an equal volume of distilled water.

Irrigation treatments were applied according to the experimental design using precise soil moisture sensors to maintain the targeted FC levels. Soil moisture was monitored and adjusted daily using a portable soil moisture sensor (Intelligent soil detector- EK572411, Guangdong, China) and verified gravimetrically by weighing a subset of pots^39^. The 100% FC level represented the soil water content at field capacity, while the deficit levels (40% and 70% FC) were calculated and maintained accordingly. Throughout the experimental period, plants were monitored daily for visible stress symptoms to ensure that the imposed water stress remained within a mild, non-lethal range.

### Data collection

Sixty days after inoculation for each treatment, the following parameters were recorded:

### Plant growth parameters

For plant growth analysis, shoot length (SL) and roots length (RL) (cm) were measured directly. The entire shoot and root systems were then carefully separated. The fresh weight of both shoots (SFW) and roots (RFW) (g) was recorded immediately after harvesting. Subsequently, the plant materials were placed in a forced-air oven at 70 °C for 72 h until a constant weight was achieved, after which the dry weight of shoots (SDW) and roots (RDW) (g) was determined. The reduction percentage in growth parameters (reduction % over control) were calculated by the following formula: Reduction %= ((C-T) /C) x100.

Where C = Parameter value in control (C+), T= Parameter value in treatment.

### T. semipenetrans criteria

For the nematological evaluation, the root system of each plant was gently but thoroughly washed to remove soil particles. The cleaned roots were stained with acid fuchsin according to Bybd et al.(1983)^40^ to count numbers of females, eggs and egg masses per root system under a stereomicroscope. Simultaneously, a 250 g soil sample was taken from the rhizosphere of each pot for count the number of J2s using the Cobb decanting and sieving technique^41^. The nematode final population (Pf) per root calculated by the following formula:

$$Pf{\text{ }} = {\text{ }}\left( {Number{\text{ }}egg{\text{ }}masses{\text{ }} \times {\text{ }}Mean{\text{ }}eggs{\text{ }}per{\text{ }}egg{\text{ }}masse} \right){\text{ }} + {\text{ }}Females{\text{ }} + Developmental{\text{ }}stages + J2s{\text{ }}in{\text{ }}250{\text{ }}g{\text{ }}soil)$$

Pf = (Number egg masses × Mean eggs per egg masse) + Females + Developmental stages + J2s in 250 g soil).

The reproduction factor (Rf) was calculated for each treatment using the formula Rf = Pf / Pi, where Pi is the initial inoculation density of 2500 nematodes per plant. The percentage reduction in nematode Pf were calculated by the following formula^42^:

$${Reduction\% {\text{ }} = {\text{ }}\left( {\left( {Pf{\text{ }}of{\text{ }}control{\text{ }} - {\text{ }}Pf{\text{ }}of{\text{ }}treatment} \right){\text{ }}/Pf{\text{ }}of{\text{ }}control} \right)){\text{ }}x100\;}$$

### Physiological and biochemical markers

Root tissues were sampled for the analysis of key physiological and biochemical compounds, including:

### Photosynthetic pigments

Concentrations of chlorophyll a and b were measured by using spectrophotometry in accordance by approach of Lichtenthaler (1987)^43^. Fresh leaf tissue was heated to 60–70 °C until it was entirely colourless, then extracted in ethanol 95% (v/v). Following centrifugation of the extract, measurements of the supernatant absorbance were read at 663, 644, and 452 nm. Each concentration of pigment was determined using standard formulas and is given as milligrams per gram of fresh weight (mg g^− 1^ FW).

### Plant extract preparation

For the analysis of primary metabolites and enzymatic, non-enzymatic antioxidants, fresh leaf samples were homogenized in an ice-cold extraction buffer containing 50 mM Tris-HCl (pH 7.0), 1 mM sodium EDTA, and 3 mM MgCl_2_ using a pre-chilled mortar and pestle. The homogenate was centrifuged at 5,000 rpm for 10 min at 4 °C, and the resulting supernatant was collected and stored at -20 °C until subsequent biochemical analyses^44^.

### Non-enzymatic antioxidants

#### Total Flavonoids

Total flavonoid contents were determined spectrophotometrically according to the method of Zhishen et al. (1999)^45^. Briefly, fresh leaf tissue was homogenized in a methanol extraction solvent. The homogenate was centrifuged, and an aliquot of the supernatant was mixed with sodium nitrite solution, followed by the addition of aluminium chloride. The reaction was terminated by adding sodium hydroxide, and the absorbance of the resulting pink-colored solution was measured at 510 nm. The total flavonoid content was calculated using a quercetin standard curve and expressed as mg QE g^− 1^ FW.

### Total phenols

Total phenols were determined by mixing 0.1 ml sample extract with 10 drops of concentrated HCL and boiled in a water bath for 10 min, then left to cool. 1.0 ml Folin and 5 ml of 20% Na2Co_3_ were added. The mixture was diluted to 10 ml as the final volume by distilled water. Colour optical density of the reacted mixture was measured on absorbance spectrophotometer (Miltonroy Spectronic) at 520 nm after 30 min according to Snell and Snell (1953)^46^. Phenol content was determined as mg g^− 1^ FW min^− 1^.

### Osmolyte

The proline concentration was determined according to the method described by Moore and Stein (1948)^47^. 1 ml of the sample extract was reacted with acid ninhydrin reagent, glacial acetic acid and the mixture was heated in a boiling water bath for 1 h. After cooling, the chromophore was extracted using toluene, and the absorbance of the colored layer was measured at 520 nm using a spectrophotometer. The proline concentration was calculated from a standard calibration curve prepared with known concentrations of pure proline and expressed as milligrams per gram of fresh weight (mg g^− 1^ FW).

### Enzymatic antioxidants (Oxidative-stress enzymes)

#### Peroxidase (POD)

Peroxidase was assayed using photochemical approach as outlined by Amako et al. (1994)^48^. The following sequences were added to the reacted mixture: 50.0 mmol/L K-phosphate buffer (PH 7.0), 1.0 mmol/L EDTA, 0.5 mmol/L Na ascorbate, 0.25 mmol/L H2O2 and 0.1 ml enzyme solution. The absorbance increase at 430 nm was determined relative blank with phosphate buffer instead of enzyme extract. Under normal test conditions, one unit of enzyme activity was defined as the quantity of the enzyme that changed the optical density at 430 nm per minute at 25 °C. The units of specific activity were expressed in mg g^− 1^ FW min^− 1^.

### Polyphenol Oxidase (PPO)

The photochemical approach, as outlined by Coseteng and Lee (1987)^49^, was used to determine polyphenol oxidase. The following sequences were added to the reacted mixture: 0.25 ml of 00.9 M catechol, 200mM potassium buffer, pH 6.2, and 0.1 ml of enzyme extract. At 420 nm and at 30 °C, the rise in absorbance was detected. The quantity of an enzyme that raises absorbance by 0.001 units per minute at 25 °C is referred to as one unit of enzyme activity (min^− 1^ g^− 1^ FW).

#### Catalase (CAT)

The catalase activity was measured according to the description of Aebi (1974)^50^. Where, 2.9 ml of a reactive mixture comprised 0.1M H_2_O_2_ 5% and 50mM sodium phosphate buffer (pH 7.6) was mixed with 0.1 ml of enzyme extract. By tracking the decrease in absorbance at 240 nm brought on by H2O2 consumption, the activity of catalase was determined. The unit of catalase activity was mg/g/fresh weight/min. The breakdown of 1µmol of H2O2 per minute was considered to be one unit of enzyme activity (µmol H_2_O_2_ min^− 1^ g^− 1^ FW).

### Data analysis and processing

Multiple statistical parameters were used to address the multidimensional nature of the data. All experimental data were subjected to normality tests using Shapiro-Wilk test^51^ and for homogeneity of variances using Levene test^52^ prior to performing analysis of one-way analysis of variance (ANOVA) by COSTAT software package (CoHort Software). Treatment means were separated using Duncan Multiple Range Test (DMRT) at a 5% probability level (*P* ≤ 0.05)^53^, and the least significant difference (LSD _0.05_) was also calculated at the 5% probability level. In addition, the coefficient of determination (R^2^) was computed using Microsoft Excel without modification to assess the strength of these relationships^54^. Where, R^2^ value ranges between 0 and 1 (0 ≤ R^2^≤ 1); values closer to 1 indicate a meaningful relationship and a higher accuracy of the regression model in explaining the variability of the data. Finally, the calculated values were integrated to establish a regression-based model (integrated matrix)^55^ describing the interactive influence of *T. semipenetrans* reproduction dynamics on plant growth performance under tested water stress levels.

## Data Availability

All data analyzed or generated for the research are included in the article.
